# Quality of Life in Systemic Lupus Erythematosus

**DOI:** 10.1080/17402520600877760

**Published:** 2006

**Authors:** Pantelis Panopalis, Ann E. Clarke

**Affiliations:** 1 University of California UCSF Box 0920 San Francisco CA 94143-0920 USA; 2 Divisions of Clinical Immunology/Allergy and Clinical Epidemiology Department of Medicine McGill University Health Centre (Montreal General Hospital) 1650 Cedar Avenue Montreal Que. H3G-1A4 Canada

## Abstract

Systemic lupus erythematosus (SLE) is a pervasive disease with wide-ranging effects on physical, psychological and social well-being. As such, a comprehensive assessment of SLE should include several different outcomes, such as quality of life (QoL) and economic costs, in addition to measures of disease activity and damage. In fact, disease effects on QoL are often considered of greater overall importance to patients. Two approaches have been used in the measurement of QoL: generic questionnaires and disease-specific questionnaires. Generic questionnaires are designed to be used across various conditions and populations, whereas disease-specific questionnaires are designed to measure outcomes in one specific disease or condition. The most commonly used measure of QoL is the Medical Outcomes Study Short Form 36 (SF-36), which is a generic measure that is applicable in a variety of conditions, including SLE. Recently, SLE-specific measures have been developed that may prove to be more responsive than generic measures. The hope is that improved outcome measures will allow for better assessment of SLE and eventually facilitate drug development and improve patient care.


*Clinical & Developmental Immunology*, June–December 2006; 13(2–4): 321–324

### Quality of life in systemic lupus erythematosus

PANTELIS PANOPALIS[1] & ANN E. CLARKE[2]

[1]*University of California, UCSF Box 0920, San Francisco, CA 94143-0920, USA, and* [2]*Divisions of Clinical Immunology/Allergy and Clinical Epidemiology, Department of Medicine, McGill University Health Centre (Montreal General Hospital), 1650 Cedar Avenue, Montreal, Que., Canada H3G-1A4*

**Abstract**

Systemic lupus erythematosus (SLE) is a pervasive disease with wide-ranging effects on physical, psychological and social well-being. As such, a comprehensive assessment of SLE should include several different outcomes, such as quality of life (QoL) and economic costs, in addition to measures of disease activity and damage. In fact, disease effects on QoL are often considered of greater overall importance to patients. Two approaches have been used in the measurement of QoL: generic questionnaires and disease-specific questionnaires. Generic questionnaires are designed to be used across various conditions and populations, whereas disease-specific questionnaires are designed to measure outcomes in one specific disease or condition. The most commonly used measure of QoL is the Medical Outcomes Study Short Form 36 (SF-36), which is a generic measure that is applicable in a variety of conditions, including SLE. Recently, SLE-specific measures have been developed that may prove to be more responsive than generic measures. The hope is that improved outcome measures will allow for better assessment of SLE and eventually facilitate drug development and improve patient care.

**Keywords:** *Systemic lupus erythematosus, quality of life, outcome measures, drug development*

### Introduction

SLE is a pervasive disease that results in variable and occasionally life-threatening, manifestations. It afflicts young people disproportionately, often at a crucial time in their lives when they are trying to establish relationships, start families and launch careers. As a result, persons with SLE may experience a wide range of physical, psychological and social problems that are not always fully captured by descriptions of the disease's physiological consequences alone. In order to characterize the full spectrum of the effects of SLE, a comprehensive assessment should consider a variety of other outcomes, which may be of equal or even greater importance to the patient. One such outcome is QoL, which is increasingly being recognized as an important aspect of chronic diseases and considered by many as a relevant measure of efficacy in clinical trials.

### Outcome measures used in SLE

The course of SLE is characterized by exacerbations (or flares) of disease activity and disease damage. Disease damage is permanent and may result from repeated flares of disease activity, or as a result of adverse effects of treatments or other co-morbidities. Measures of disease activity include the systemic lupus activity measure-revised (SLAM-R) (Bae et al. 2001), the SLE disease activity index (SLEDAI) (Bombardier et al. 1992) and the British Isles Lupus Assessment Group (BILAG) disease activity index (Stoll et al. 1996). Disease damage is most often measured through the Systemic Lupus International Collaborating Clinics/American College of Rheumatology Damage Index (SLIC/ACR DI) (Gladman et al. 1996, 1997). However, in addition to disease activity and damage, there are other important consequences of disease that include changes in QoL, employment and social functioning. Therefore, in an effort to improve assessment of outcomes in SLE, the outcome measures in rheumatology clinical trials (OMERACT) group has recommended that trials of SLE include outcome measures of QoL, adverse events and economic costs, in addition to measures of disease activity and disease damage (Strand et al. 2000).

Correspondence: P. Panopalis, University of California, UCSF Box 0920, San Francisco, CA 94143-0920, USA. Tel: 1 415 476 3774. Fax: 1 514 934 8293. E-mail: pantelis.panopalis@mail.mcgill.ca

ISSN 1740-2522 print/ISSN 1740-2530 online © 2006 Taylor & Francis
DOI: 10.1080/17402520600877760

### Measurement of quality of life

QoL and more specifically, HRQoL refers to the impact that a disease and its treatment has on an individual's ability to function and his or her perceived well-being in physical, mental and social domains of life. Increasing pressure on the use of health care resources has resulted in a need for measures that will best assess the relative effectiveness and appropriateness of rival medical treatments. Measurement of HRQoL, in addition to more objective clinical indicators of disease, allows for a more comprehensive assessment and in some cases may prove to be a more sensitive indicator of treatment response than measures of disease activity or damage (Strand et al. 2003). Furthermore, information about broader patient outcomes, including outcomes of importance to patients, helps physicians and patients when making decisions about the most appropriate health care. The challenge remains to identify instruments that will accurately and reliably assess these disease outcomes.

Measurement of HRQoL has traditionally relied on two basic approaches: the use of *generic questionnaires* and the use of *disease-specific questionnaires*. Generic questionnaires were developed for general use and may be used in a variety of diseases and populations. They allow for comparison with other groups and other conditions and allow measurement of dysfunction for individuals experiencing more than one condition. In contrast, disease-specific questionnaires are designed to measure outcomes in a specific disease. Because they incorporate elements specific to particular diseases, they are believed to be more responsive than generic instruments. Only recently have disease-specific instruments been developed for use in SLE and these are not yet in wide use.

### *Generic measures (Table I)*

At present, the most commonly used measure of HRQoL is the SF-36. Developed by Ware et al. (1992), the SF-36 is a generic, 36-item self-report questionnaire designed to be used in a variety of conditions, populations, and settings. It includes eight subscales (physical functioning, social functioning, role limitations due to physical problems, role limitations due to emotional problems, mental health, energy/vitality, pain and general health perception) that can be summarized into two component scores: the physical component summary score and the mental component summary score. The SF-36 has been shown to be a valid and reliable instrument in SLE (Stoll et al. 1997) and has been used in numerous studies in SLE. Using the SF-36, several studies have demonstrated that persons with SLE have a significantly poorer QoL than persons without a chronic illness (Stoll et al. 1997; Sutcliffe et al. 1999).

Other generic HRQoL questionnaires that have been used in SLE include the European QoL scale (EQ-5D) (The EuroQol Group 1990; Kind 1996), the World Health Organization quality of life scale (WHOQOL-Bref) (The WHOQOL Group 1998), the Nottingham health profile (NHP) (Hunt et al. 1981) and the sickness impact profile (SIP) (Bergner et al. 1981). The EQ-5D is a simple measure that assesses five dimensions of health status: mobility, self-care, usual activities, pain/discomfort, and anxiety/depression. Wang et al. (2001) in a study of 54 persons with SLE, showed the EQ-5D to be a valid instrument for the measurement of HRQoL. Luo et al. (2003a,b) have shown both a Singaporean English and a Singaporean Chinese version of the EQ-5D to be valid in persons with various rheumatic diseases, including SLE. The WHOQOL-Bref, a 26 item questionnaire assessing four domains of QoL (physical, psychological, social and environmental) was evaluated in 73 patients from India (Khanna et al. 2004). Only the physical and psychological domains of QoL were found to be impaired in patients with active SLE. The NHP and the SIP have been used in a variety of diseases; however, neither has been validated in SLE.

### *Disease-specific measures*

The Stanford health assessment questionnaire (HAQ) (Fries et al. 1980) was initially developed for use in persons with arthritis and has become the most commonly used measure of functioning in the rheumatic diseases, particularly rheumatoid arthritis. Although initially developed to assess the impact of arthritis, it has been used and validated in a variety of other conditions and thus may also be regarded as a generic instrument. The HAQ is a 20-item questionnaire that assesses activities of daily living in eight domains: dressing, arising, eating, walking, hygiene, reaching, gripping and errands and chores. Although it has been shown to be a valid instrument for use in SLE (Hochberg and Sutton 1988; Milligan et al. 1993), an important limitation is that it only assesses physical functioning. Therefore, for a more comprehensive assessment, it should be used in combination with instruments that also assess psychosocial functioning.

Another instrument that was developed for use mainly in persons with arthritis is the arthritis impact measurement scale (AIMS) (Meenan et al. 1982), which was revised in 1992 (AIMS2) (Meenan et al. 1992). It is a 78-item questionnaire that assesses physical functioning, activities of daily living, social activities, social support, arthritis pain, work, level of tension, mood, satisfaction with health, general health perceptions, overall impact of arthritis and medications. In SLE, the AIMS has been used in only one

Table I. Health-related QoL questionnaires used in SLE.

| Questionnaire | Domains assessed | | Number of items |
|---|---|---|---|
| | Physical/functional impact | Social/emotional impact | |
| Generic | | | |
| SF-36 | Yes | Yes | 36 |
| EQ-5D | Yes | Yes | 5 (also contains a visual analog scale) |
| WHOQOL-Bref | Yes | Yes | 26 |
| NHP | Yes | No | 38 |
| SIP | Yes | Yes | 136 |
| Disease-specific | | | |
| HAQ | Yes | No | 20 |
| AIMS | Yes | Yes | 78 |
| SLE-specific | | | |
| SLEQOL | Yes | Yes | 40 |
| SSC | Yes | Yes | 38 |
| LupusQoL | Yes | Yes | 34 |

study that compared patients with SLE and rheumatoid arthritis (Burckhardt et al. 1993).

### *SLE-specific measures*

Three disease-specific QoL measures have been recently developed for use in SLE.

Leong et al. (2005) developed and validated a new 40-item SLE-specific QoL instrument, the systemic lupus erythematosus-specific quality-of-life (SLE-QOL). The questionnaire consists of 6 subsections: physical functioning, activities, symptoms, treatment, mood and self-image. It was evaluated in 275 persons with SLE and was found to be more responsive to change than the SF-36. It was shown to be valid, possessing construct validity, face and content validity, internal consistency, test–retest reliability and responsiveness. Another SLE-QoL questionnaire was recently developed by Grootscholten et al. (2003) the SSC. Testing for reliability and reproducibility has shown satisfactory internal consistency and test–retest reliability. A third SLE-specific measure of HRQoL is the Lupus QoL Scale (LupusQoL) (Teh et al. 2005). This is a 34-item questionnaire that assesses 8 domains: physical functioning, pain, emotional functioning, fatigue, body image, sex, planning and burden to others. It possesses internal consistency, test–retest reliability and concurrent validity when compared with the SF-36.

The development of these SLE-specific instruments may prove to be invaluable in SLE drug trials, which have suffered from a lack of sensitive outcome measures that are able to detect clinically meaningful differences between competing therapeutic strategies. These questionnaires may prove to be more responsive in certain situations and may address issues of particular concern in persons with SLE. Nevertheless, they will require further evaluation before they can be recommended for routine use. Furthermore, although there are advantages to using disease-specific questionnaires, the current general consensus is that generic measures should be used preferentially, supplemented with disease-specific measures where applicable.

### Conclusion

The various questionnaires described above have been used in the assessment of a number of diseases, including SLE. Each has its own advantages and disadvantages and the choice of which measure to use should be made on an individual basis, taking into consideration the specific aims of the study. Generic measures are widely used and have the advantage of allowing comparisons between different conditions. The SF-36, in particular, is currently the preferred outcome measure for HRQoL in US health policy research. It has also proven to be more responsive than measures of disease activity in at least one clinical trial of a novel therapeutic agent for SLE, LPJ-394 (Strand et al. 2003). The hope is that newly developed SLE-specific questionnaires will be even more responsive, so as to better assess the efficacy of current and new therapeutic agents. No new drug has been approved for the treatment of SLE in over 25 years. This failure to demonstrate benefit may be at least partly due to a lack of appropriate outcome measures. Better measures of HRQoL, in combination with improved measures of disease activity and disease damage, will help drive the development of new therapeutic agents and facilitate their approval.

### References

Bae SC, Koh HK, Chang DK, Kim MH, Park JK, Kim SY. 2001. Reliability and validity of systemic lupus activity measure-revised (SLAM-R) for measuring clinical disease activity in systemic lupus erythematosus. Lupus 10(6):405–409.

Bergner M, Bobbitt RA, Carter WB, Gilson BS. 1981. The sickness impact profile: Development and final revision of a health status measure. Med Care 19(8):787–805.

Bombardier C, Gladman DD, Urowitz MB, Caron D, Chang C. The committee on prognosis studies in SLE 1992. Derivation of the SLEDAI. A disease activity index for lupus patients. Arthritis Rheum 35:630–640.

Burckhardt CS, Archenholtz B, Bjelle A. 1993. Quality of life of women with systemic lupus erythematosus: A comparison with women with rheumatoid arthritis. J Rheumatol 20(6):977–981.

Fries JF, Spitz PW, Kraines RG, Holman HR. 1980. Measurement of patient outcome in arthritis. Arthritis Rheum 23:127–134.

Gladman DD, Ginzler EM, Goldsmith CH, Fortin PR, Liang MH, Urowitz MB, et al. 1996. The development and initial validation of the Systemic Lupus International Collaborating Clinics/American College of Rheumatology Damage Index for systemic lupus erythematosus. Arthritis Rheum 39:363–369.

Gladman DD, Urowitz MB, Goldsmith CH, Fortin PR, Ginzler EM, Gordon C, et al. 1997. The reliability of the Systemic Lupus International Collaborating Clinics/American College of Rheumatology Damage Index in patients with systemic lupus erythematosus. Arthritis Rheum 40:809–813.

Grootscholten C, Ligtenberg G, Derksen RHWM, Schreurs KMG, Glas-Vos JW, Hagen EC, et al. 2003. Health-related quality of life in patients with systemic lupus erythematosus: Development and validation of a lupus specific symptom checklist. Qual Life Res 12(6):635–644.

Hochberg MC, Sutton JD. 1988. Physical disability and psychosocial dysfunction in systemic lupus erythematosus. J Rheumatol 15(6):959–964.

Hunt SM, McKenna SP, McEwen J, Williams J, Papp E. 1981. The Nottingham health profile: Subjective health status and medical consultations. Soc Sci Med [A] 15(3 Pt 1):221–229.

Khanna S, Pal H, Pandey RM, Handa R. 2004. The relationship between disease activity and quality of life in systemic lupus erythematosus. Rheumatology (Oxford) 43(12):1536–1540.

Kind P. 1996. The Euroqol instrument: An index of health-related quality of life. In: Spiker B, editor. Quality of life and pharmacoeconomics in clinical trials. Philadelphia, PA: Lippincott-Raven Publishers. p 191–201.

Leong KP, Kong KO, Thong BY, Koh ET, Lian TY, Teh CL, et al. 2005. Development and preliminary validation of a systemic lupus erythematosus-specific quality-of-life instrument (SLEQOL). Rheumatology 44(10):1267–1276.

Luo N, Chew LH, Fong KY, Koh DR, Ng SC, Yoon KH, et al. 2003a. Validity and reliability of the EQ-5D self-report questionnaire in English-speaking Asian patients with rheumatic diseases in Singapore. Qual Life Res 12(1):87–92.

Luo N, Chew LH, Fong KY, Koh DR, Ng SC, Yoon KH, et al. 2003b. Validity and reliability of the EQ-5D self-report questionnaire in Chinese-speaking patients with rheumatic diseases in Singapore. Ann Acad Med Singapore 32(5):685–690.

Meenan RF, Gertman PM, Mason JH, Dunaif R. 1982. The arthritis impact measurement scales. Further investigations of a health status measure. Arthritis Rheum 25(9):1048–1053.

Meenan RF, Mason JH, Anderson JJ, Guccione AA, Kazis LE. 1992. AIMS2. The content and properties of a revised and expanded arthritis impact measurement scales health status questionnaire. Arthritis Rheum 35(1):1–10.

Milligan SE, Hom DL, Ballou SP, Persse LJ, Svilar GM, Coulton CJ. 1993. An assessment of the health assessment questionnaire functional ability index among women with systemic lupus erythematosus. J Rheumatol 20(6):972–976.

Stoll TS, Stucki G, Malik J, Pyke S, Isenberg DA. 1996. Further validation of the BILAG disease activity index in patients with systemic lupus erythematosus. Ann Rheum Dis 55(10): 756–760.

Stoll T, Gordon C, Seifert B, Richardson K, Malik J, Bacon PA, et al. 1997. Consistency and validity of patient administered assessment of quality of life by the MOS SF-36; its association with disease activity and damage in patients with systemic lupus erythematosus. J Rheumatol 24:1608–1614.

Strand V, Gladman D, Isenberg D, Petri M, Smolen J, Tugwell P. 2000. Endpoints: Consensus recommendations from OMERACT IV. Outcome measures in rheumatology. Lupus 9(5):322–327.

Strand V, Aranow C, Cardiel MH, Alarcon-Segovia D, Furie R, Sherrer Y, et al. 2003. Improvement in health-related quality of life in systemic lupus erythematosus patients enrolled in a randomized clinical trial comparing LJP 394 treatment with placebo. Lupus 12(9):677–686.

Sutcliffe N, Clarke AE, Levinton C, Frost C, Gordon C, Isenberg DA. 1999. Associates of health status in patients with SLE. J Rheumatol 26:2352–2356.

Teh L, McElhone K, Bruce IN, et al. 2005. Development and validation for a disease specific quality of life measure for adults with systemic lupus erythematosus, the LupusQoL. Arthritis and Rheum 52(Suppl. 9):S188.

The EuroQol Group 1990. EuroQol: A new facility for the measurement of health-related quality of life. Health Policy 16:199–208.

The WHOQOL Group 1998. Development of the World Health Organization WHOQOL-BREF quality of life assessment. Psychol Med 28(3):551–558.

Wang C, Mayo NE, Fortin PR. 2001. The relationship between health related quality of life and disease activity and damage in systemic lupus erythematosus. J Rheumatol 28(3):525–532.

Ware JE, Jr, Sherbourne CD. 1992. The MOS36 item short-form health survey (SF-36). I. Conceptual framework and item selection. Med Care 30:473–483.

